# Supplementary material for: Long Noncoding RNA LINC02470 Sponges MicroRNA-143-3p and Enhances SMAD3-Mediated Epithelial-to-Mesenchymal Transition to Promote the Aggressive Properties of Bladder Cancer
Source: Cancers (Basel). 2022 Feb 15;14(4):968. doi: 10.3390/cancers14040968 (PMC8870681; doi:10.3390/cancers14040968)
Supplement: Supplementary file 1 [file cancers-14-00968-s001.zip › cancers-1584275-supplementary.pdf]

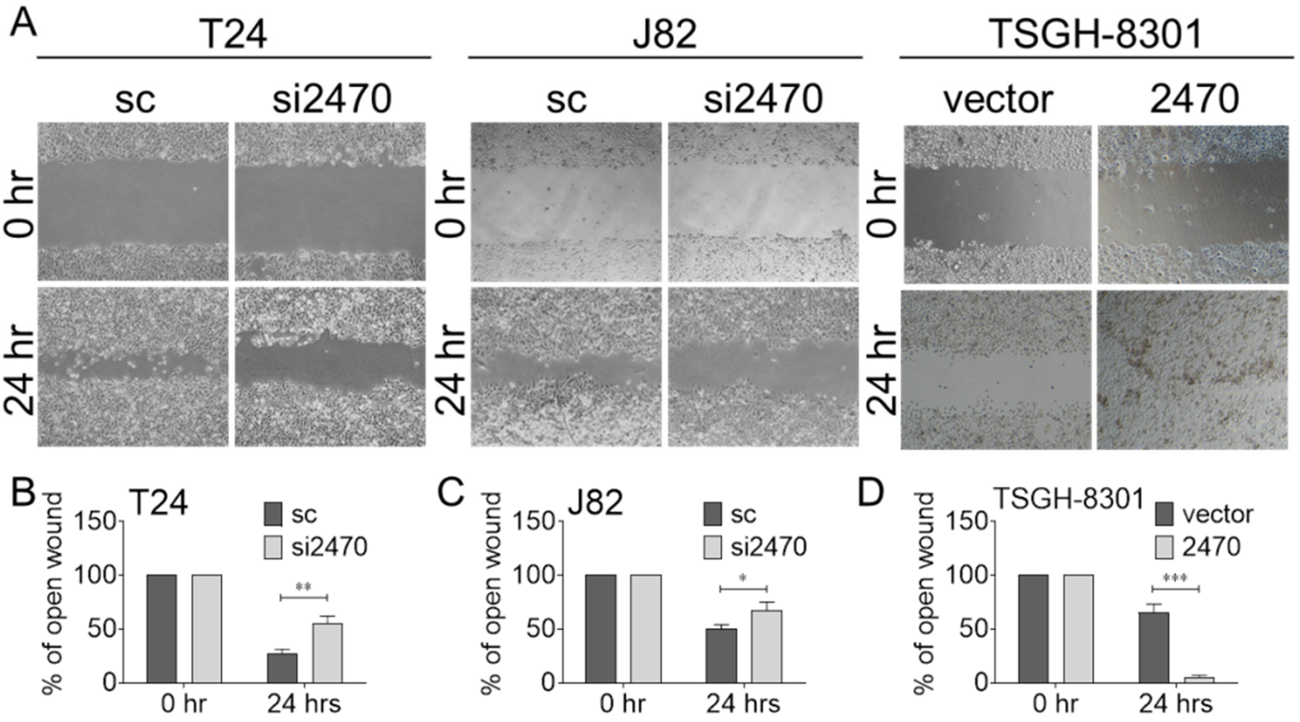

Supplemental Figure S1. (A) The wound-healing assay was used to evaluate LINC02470 effect on cell migration, and T24 and J82 cells transfected with si2470 revealed slower wound-healed ability compared to sc groups. And TSGH-8301 cells transfected with pCMV-LINC02470 (2470) revealed faster wound-healed ability compared to vector group. (B, C, D) The bar-charts were used to show the relative wound area of each group.

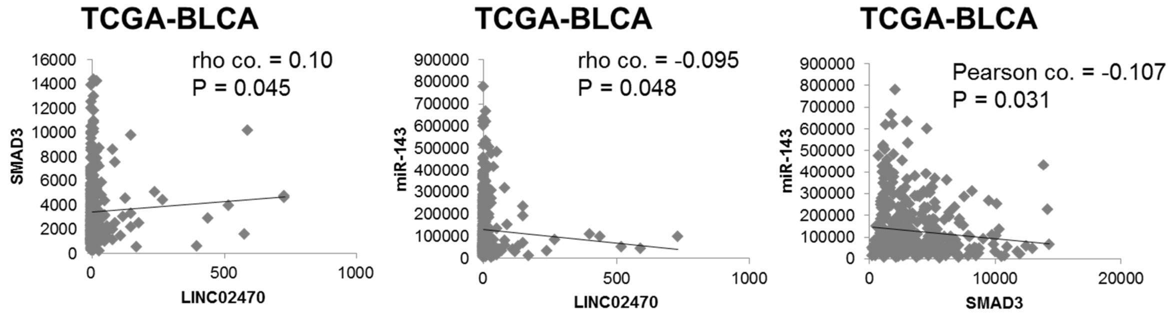

Supplemental Figure S2. The expression of SMAD3 and LINC02470 revealed a positive correlation but the expression of miR-143 was negatively correlated with either LINC02470 or SMAD3 in TCGA-BLCA dataset. The LINC02470 data were collected with discrete percentage and more proper to be analyzed with Spearman rho correlation but miR-143 and SMAD3 were recorded as continuous variables and analyzed with Pearson correlation.

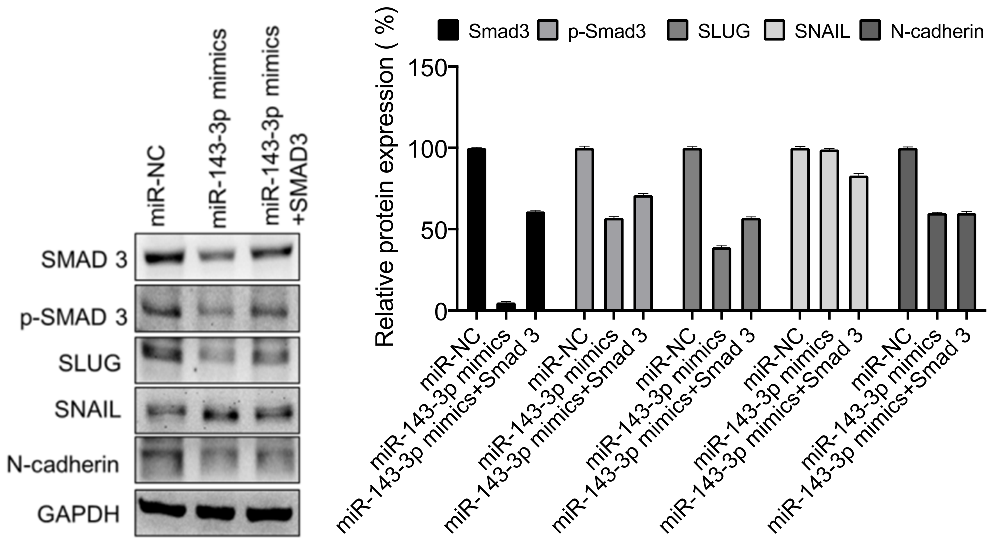

Supplemental Figure S3. The miR143-3p mimics significantly inhibited SMAD3, active phosphorylated SMAD3, SLUG and N-cadherin. And overexpressed SMAD3 rescued the inhibitory effects of miR-143-3p mimics and recovered the expression of SMAD3, active phosphorylated SMAD3, SLUG and N-cadherin. And Bar chart showing the relative protein levels, which were normalized to GAPDH; the miR-NC group was used as the comparative baseline.

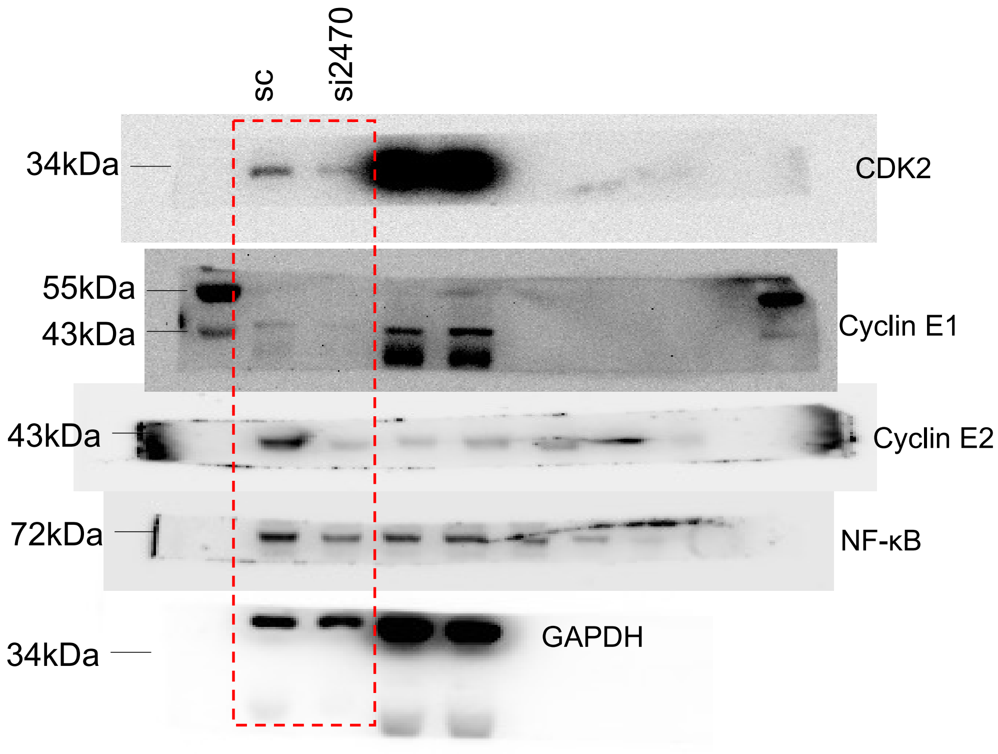
sc
si2470
34kDa
CDK2
55kDa
43kDa
Cyclin E1
43kDa
Cyclin E2
72kDa
NF-κB
34kDa
GAPDH

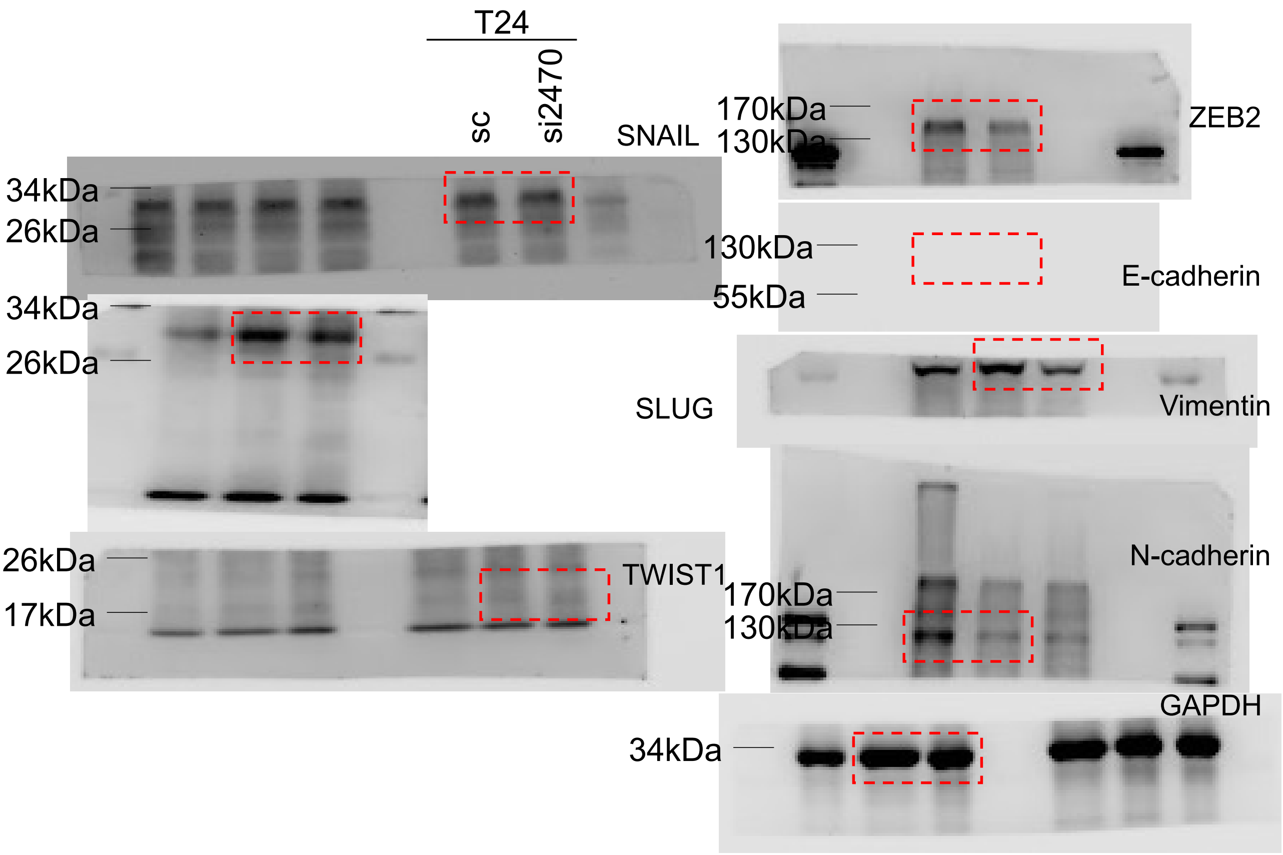
T24
sc
si2470
SNAIL
34kDa
26kDa
34kDa
26kDa
SLUG
26kDa
17kDa
TWIST1
170kDa
130kDa
ZEB2
130kDa
55kDa
E-cadherin
Vimentin
N-cadherin
170kDa
130kDa
GAPDH
34kDa

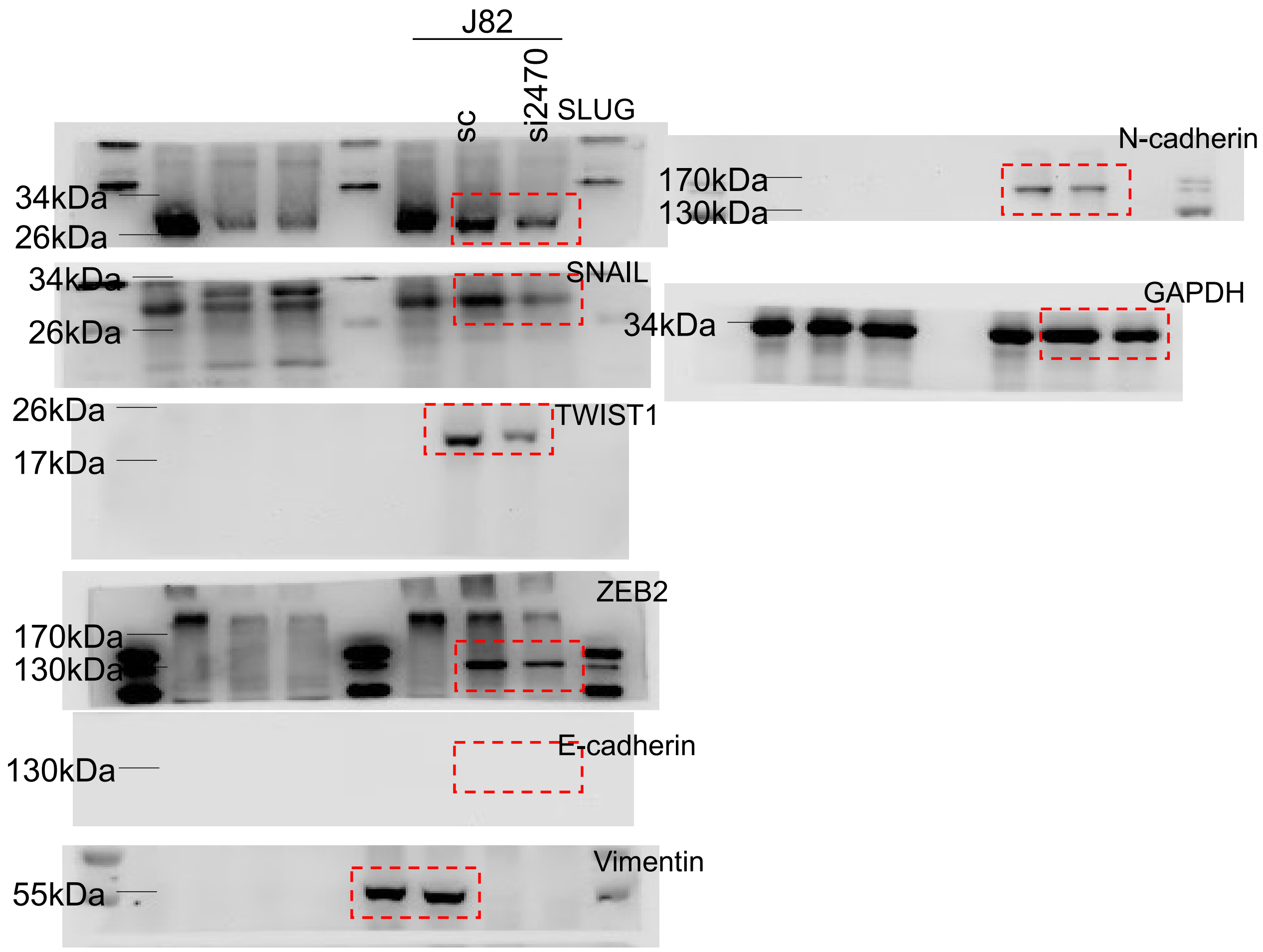

J82
sc
si2470
SLUG
34kDa
26kDa
SNAIL
34kDa
26kDa
TWIST1
26kDa
17kDa
ZEB2
170kDa
130kDa
E-cadherin
130kDa
Vimentin
55kDa
N-cadherin
170kDa
130kDa
GAPDH
34kDa

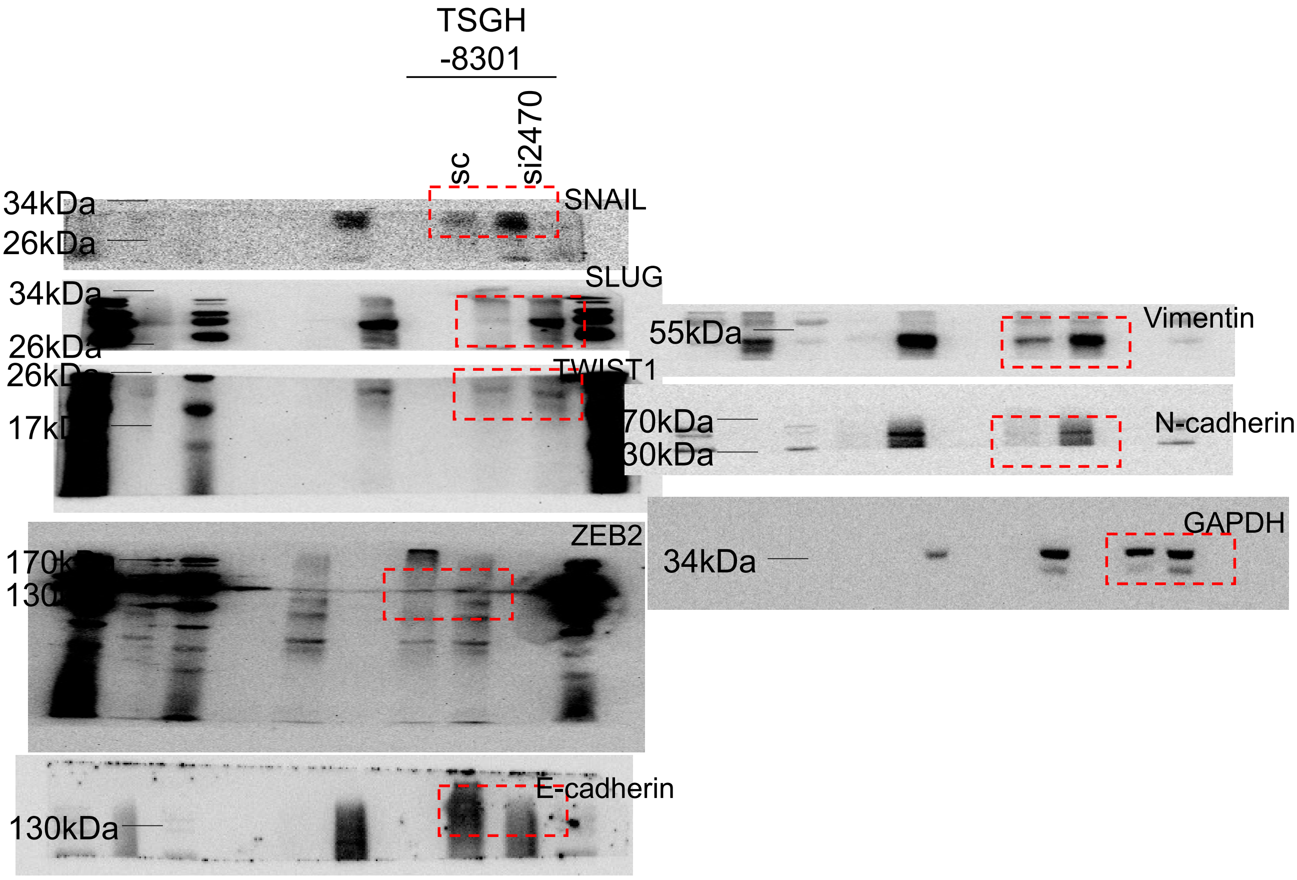
TSGH
-8301
sc
si2470
34kDa
26kDa
SNAIL
34kDa
26kDa
SLUG
26kDa
TWIST1
ZEB2
130kDa
E-cadherin
55kDa
Vimentin
N-cadherin
34kDa
GAPDH

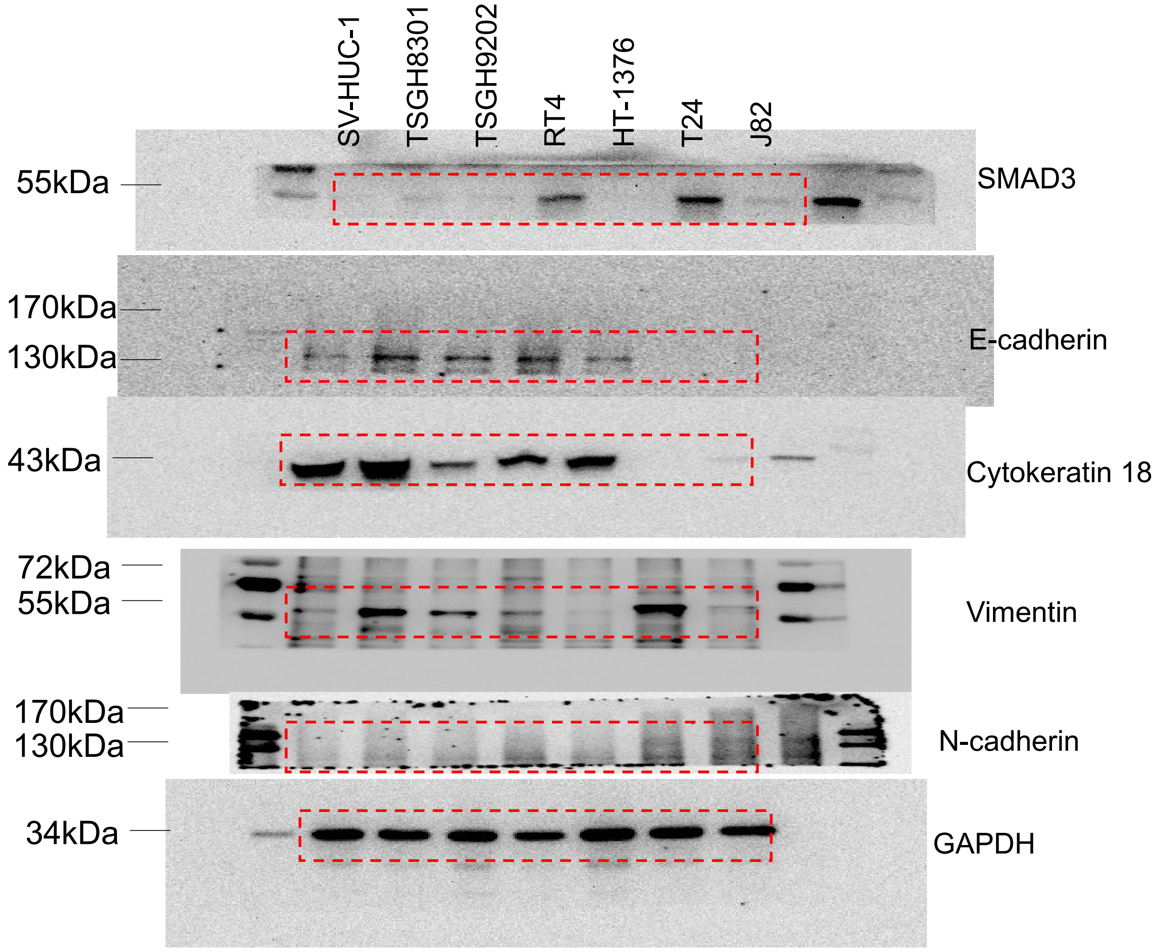

SV-HUC-1
TSGH8301
TSGH9202
RT4
HT-1376
T24
J82
55kDa
SMAD3
170kDa
130kDa
E-cadherin
43kDa
Cytokeratin 18
72kDa
55kDa
Vimentin
170kDa
130kDa
N-cadherin
34kDa
GAPDH

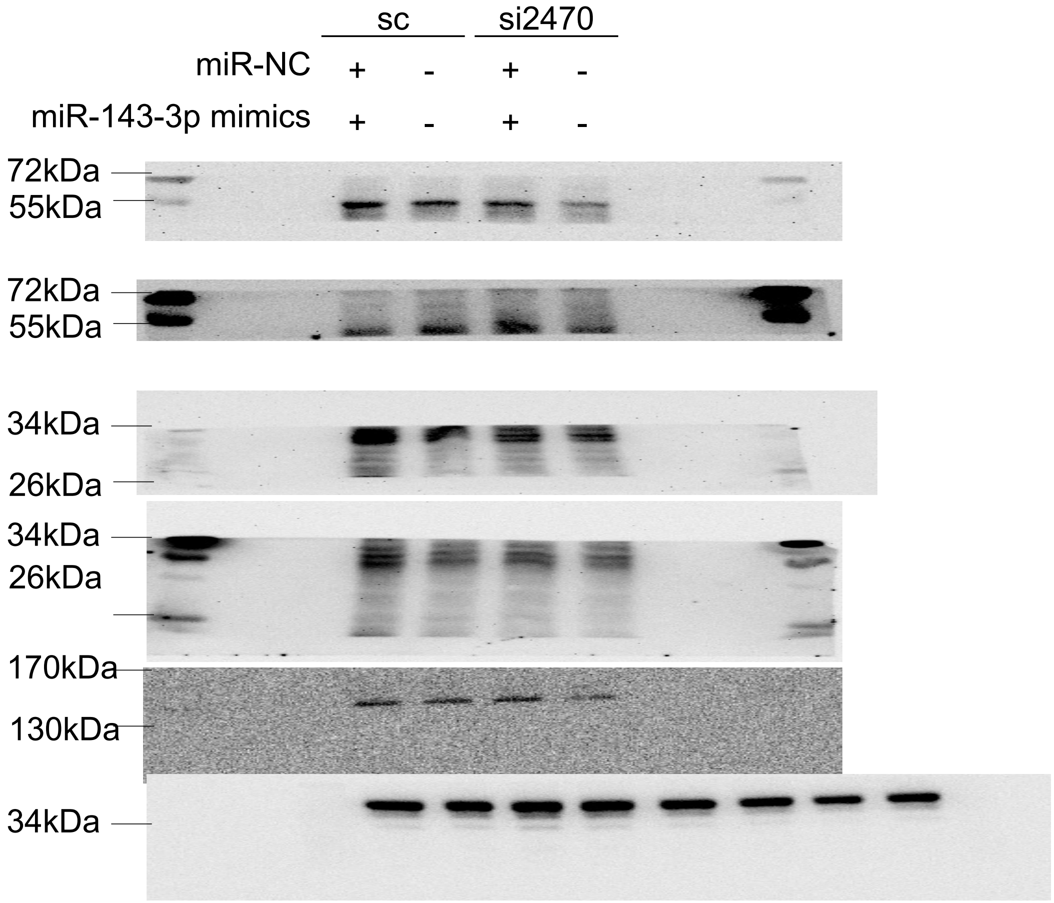

sc
si2470
miR-NC + - + -
miR-143-3p mimics + - + -
72kDa
55kDa
72kDa
55kDa
34kDa
26kDa
34kDa
26kDa
170kDa
130kDa
34kDa

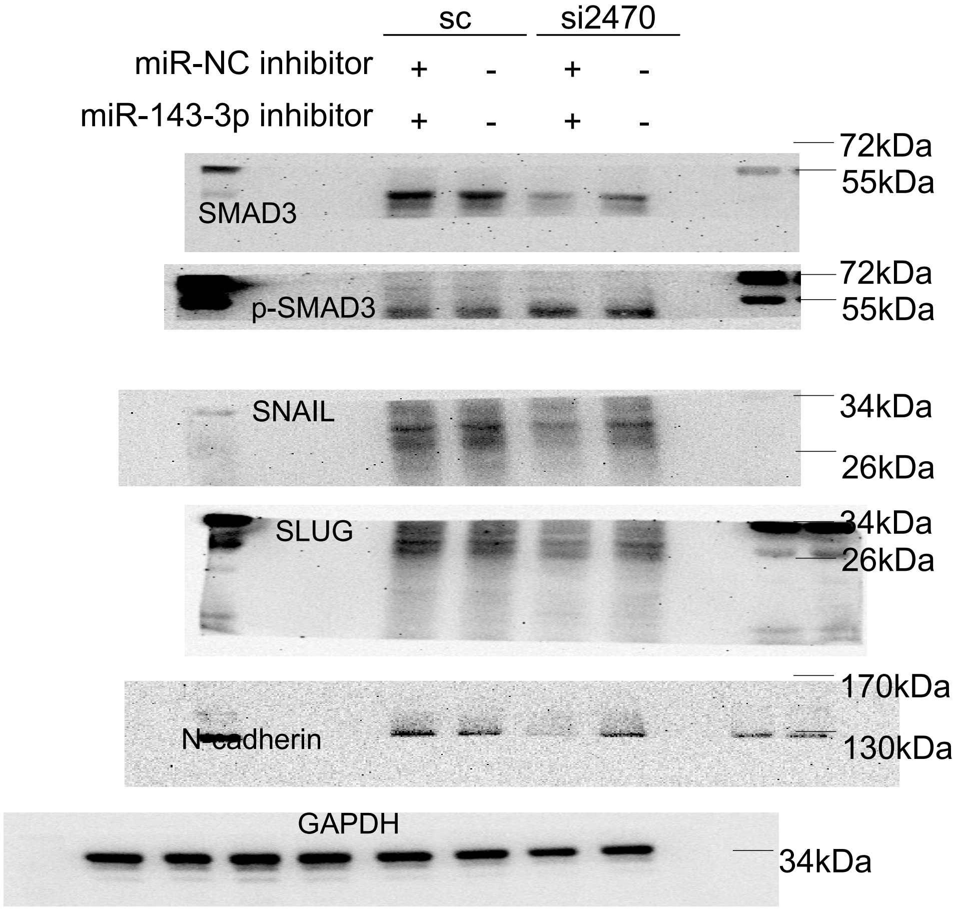

Supplementary Figure S4: Full Western blot images

Supplementary Table S1. Primer list

MIR4697HG	Forward (5′-3′)	CCACGGTCTCTGCACAAGGG
	Reverse (5′-3′)	TGCATGTGGCCAGAAGTCGG
TRMU	Forward (5′-3′)	GGGGTCTGTACTGCCGACAA
	Reverse (5′-3′)	GCATCCTGGGAAACCTGGCATT
IGFL2-AS1	Forward (5′-3′)	GCCCCAAGGGCTCATTCACA
	Reverse (5′-3′)	TGTGTAAGTAACACGTCCTGGGT
LINC01451	Forward (5′-3′)	TGAGCTGTGCATCCCGTGTG
	Reverse (5′-3′)	CGCCACAGTCCGTGTGTGAT
GLIDR	Forward (5′-3′)	CGAATCCACCCATTGCCCGT
	Reverse (5′-3′)	GAGGCGTCTCTTCCGTGCAG
LOC728673	Forward (5′-3′)	TGACGGGCGAGGGTTACTGT
	Reverse (5′-3′)	CCGCTCAGGCTCAAGAGCAA
LINC01291	Forward (5′-3′)	GTGACCAGCAGCATGTGTTTCCA
	Reverse (5′-3′)	CAGGACTCACTGGCAATTCACCT

LINC02470	Forward (5′-3′)	AGCAGACCAACACGCCAGAA
	Reverse (5′-3′)	AGAAGAAAAGCAATGCCTCCACA
NOTCH1	Forward (5′-3′)	GGTGAACTGCTCTGAGGAGATC
	Reverse (5′-3′)	GGATTGCAGTCGTCCACGTTGA
NOTCH4	Forward (5′-3′)	TTCCACTGTCCTCCTGCCAGAA
	Reverse (5′-3′)	TGGCACAGGCTGCCTTGGAATC
CTNNB1	Forward (5′-3′)	CACAAGCAGAGTGCTGAAGGTG
	Reverse (5′-3′)	GATTCCTGAGAGTCCAAAGACAG
TCF4	Forward (5′-3′)	GCCTCTTCACAGTAGTGCCATG
	Reverse (5′-3′)	GCTGGTTTGGAGGAAGGATAGC
SMAD2	Forward (5′-3′)	GGGTTTTGAAGCCGTCTATCAGC
	Reverse (5′-3′)	CCAACCACTGTAGAGGTCCATTC
SMAD3	Forward (5′-3′)	TCACTGGTGCTGGGGTTAGG
	Reverse (5′-3′)	GAAGCGAGCGGCGGCAATAA
SMAD4	Forward (5′-3′)	CTACCAGCACTGCCAACTTTCC
	Reverse (5′-3′)	CCTGATGCTATCTGCAACAGTCC
GAPDH	Forward (5′-3′)	CCTGCCGGTGACTAACCCTG
	Reverse (5′-3′)	TCCACCACTGACACGTTGGC
LINC02470-miR-143-3p-fragment	Forward (5′-3′)	CTCGTGAGGGTTGGAGATGG
	Reverse (5′-3′)	ATTCAGTTCCAGCCTTGTGGA
SMAD3-3′UTR	Forward (5′-3′)	CACCTTCCAAGGAGCCAACT
	Reverse (5′-3′)	CCCTCATGTGTGCTCTTCCT
SNAIL ChIP	Forward (5′-3′)	CAGGTGACCCGCCTCTTAAC
	Reverse (5′-3′)	AGGGTAGCTTCTGGTCCAGT
SLUG ChIP	Forward (5′-3′)	TGCCACTTCCAAATATAGGCTCTCA
	Reverse (5′-3′)	CTGGGACAGCTGTGAACAGA
